# Autism Spectrum Social Stories in Schools Trial 2 (ASSSIST‐2): a pragmatic randomised controlled trial of the Social Stories™ intervention to address the social and emotional health of autistic children in UK primary schools

**DOI:** 10.1111/camh.12740

**Published:** 2024-12-17

**Authors:** Barry Wright, Jane E. Blackwell, Kerry J. Bell, Catarina Teige, Laura Mandefield, Han‐I Wang, Charlie Welch, Arabella Scantlebury, Judith Watson, Dean McMillan, Emma Standley, Leah Attwell, Hayley Carrick, Amelia Taylor, Olivia Taylor, Rachel Hodkinson, Hannah Edwards, Hannah Pearson, Steve Parrott, David Marshall, Danielle Varley, Rebecca Hargate, Anne Mclaren, Catherine Hewitt

**Affiliations:** ^1^ Department of Health Sciences University of York York UK; ^2^ Hull York Medical School University of York York UK; ^3^ The Child Oriented Mental health Innovation Collaborative Leeds and York Partnership NHS Foundation Trust York UK; ^4^ Patient and public involvement representative

**Keywords:** Autism, school children, social skills, behaviour, social stories

## Abstract

**Background:**

Autistic children can experience mental health, social and emotional difficulties. Carol Gray's Social Stories™ are a highly personalised intervention that provide social information in a short individually tailored story.

**Methods:**

A multi‐site pragmatic cluster randomised controlled trial to evaluate the clinical and cost‐effectiveness of Social Stories™ alongside care as usual in autistic children aged 4–11 years. The primary outcome was the Social Responsiveness Scale‐2 completed by teachers 6 months post‐randomisation, analysed on an intention‐to‐treat basis. Trial Registration: ISRCTN11634810.

**Results:**

Eighty‐seven schools, including 249 children, were randomised (intervention 44 schools with 129 children, and usual care 43 schools with 120 children). After 6 months, a reduction of 1.61 points was found on the Social Responsiveness Scale‐2 in the intervention group (95% CI −4.18 to 0.96, *p* = .220) and for those who attended at least six sessions a reduction of 3.37 points (CACE 95% CI −6.65 to −0.10, *p* = .043). Children in the intervention group met their individual socio‐emotional goal more frequently than children receiving usual care alone and this was statistically significant. No statistically significant differences were found in other secondary outcomes including anxiety, depression, general health or parental stress.

**Conclusions:**

Social Stories™ represent a low‐cost, low‐burden intervention. Benefits are seen in individual socio‐emotional goals but without clinically evident impact on social responsiveness, anxiety, depression, parental stress or general health.

**Key Practitioner Message**

**What is known?**

- Social Stories^TM^ provide children with social information. The intervention involves parents and teachers (and where possible children) discussing the needs of the child.

**What is new?**

- This was the first fully powered pragmatic cluster randomised trial evaluating the effectiveness of Carol Gray's Social Stories^TM^ for improving social responsiveness in autistic children.
- Social Stories^TM^ are a low‐cost, low‐burden intervention to support autistic children within schools and at home.

**What is significant for clinical practice?**

- Despite individual Stories having limited evident impact on global social skills, Social Stories^TM^ can be useful to support children in achieving specific socio‐emotional goals.

## Introduction

Autism is a lifelong neurodevelopmental condition characterised by differences in social communication and interaction, and restricted, repetitive patterns of behaviour and interests (Lai, Lombardo, & Baron‐Cohen, 2014). Around one in 57 (1.76%) children in England are autistic (Roman‐Urrestarazu et al., 2021) and the cost of supporting these children is estimated to be between three and four times that of a typically developing child (Barrett et al., 2015). Autistic individuals require varying levels of support from different services (such as the NHS, schools, local authorities and charitable organisations). They can experience difficulties in relation to quality of life, relationships, employment and standards of living owing to the difficulty trying to navigate a neurotypical world (Knapp, Romeo, & Beecham, 2009).

The educational environment can present specific challenges for autistic children (Simpson, de Boer‐Ott, & Smith‐Myles, 2003) such as busy classroom environments, changes in school routines and difficulties navigating peer relationships. These were particularly prevalent during the COVID‐19 pandemic, impacting autistic children more negatively than neurotypical children (Morris, Hope, Foulsham, & Mills, 2023). There is a need for low‐cost, child‐friendly and evidence‐based interventions that can be delivered in community settings, such as schools, that may potentially reduce the need for specialist referral to health and education services.

This study explores whether one promising intervention could be effective in supporting autistic children in primary school. Carol Gray's Social Stories™ are a highly personalised intervention aiming to share accurate, meaningful social information about a particular goal or topic that the child needs support with in a positive and reassuring way in the form of a short story (Williams & Wright, 2016). Social Stories™ can be written and delivered both in school and at home and whilst requiring careful construction, need minimal input from experts. Previous studies have suggested that following exposure to tailored Social Stories™, autistic children have shown improvements in managing mealtime difficulties (Bledsoe, Smith, & Simpson, 2003), making independent choices and playing appropriately (Barry & Burlew, 2004), reducing anxiety (O'Connor, 2009), supporting improved communication (Adams, Gouvousis, VanLue, & Waldron, 2004), managing and reducing frustrations (Adams et al., 2004) and improving emotional regulation (Kuttler, Myles, & Carlson, 1998; Lorimer, Simpson, Smith Myles, & Ganz, 2002; Ozdemir, 2008).

This cluster randomised controlled trial (RCT) followed a feasibility study (Marshall et al., 2016) that demonstrated a high degree of acceptability with young people, families and schools. As outlined in the study protocol (Wright et al., 2020), the aim of this trial was to assess whether Social Stories™ alongside care as usual are clinically and cost‐effective in improving child social responsiveness and improving social and emotional health in autistic children in primary schools when compared with care as usual alone.

## Methods

### Study design

This trial was a pragmatic cluster RCT comparing Social Stories™ alongside care as usual with care as usual alone, defined as the existing support routinely provided for autistic children from educational and health services. Following the recruitment of participants and baseline data collection, participating schools were randomised (1:1) to one of the treatment groups. Participants were followed up at 6 weeks and 6 months post‐randomisation. This was a pragmatic trial because it was carried out in real‐world school settings with delivery of the intervention by school staff rather than research staff/settings. The full trial protocol is published (Wright et al., 2020). The trial was assigned the International Standard Randomised Controlled Trial Number (ISRCTN) ISRCTN11634810 on 23 April 2019. The trial is reported according to the CONSORT guidelines for cluster trials (Campbell, Elbourne, Altman, & Group, 2004). Ethical approval for the trial was obtained from the Health Sciences Research Governance Committee at the University of York on 02 July 2018 and the Health Research Authority (HRA) on 24 July 2019 and Northeast—York Research Ethics Committee (REC) on 23 July 2019 (19/NE/0237).

Elements of the trial are reported elsewhere including a process evaluation that assessed the fidelity of the programme, the views of stakeholders and barriers and facilitators to successful implementation (Blackwell et al., 2024) and an economic evaluation (Wang et al., 2024) including a cost‐utility analysis, that measured the incremental cost‐effectiveness ratio of the Social Stories™ intervention over and above the control arm.

### Participants

Participants were assessed for eligibility by a research assistant against the following criteria: aged 4–11 years, with a clinical diagnosis of autism spectrum disorder (which was also recorded on their school needs register), and had socio‐emotional learning needs identified by their teachers or parents that created daily challenges for them. All children included in the study attended a participating primary school or special educational needs (SEN) school within Yorkshire and the Humber. Parents/guardians were able to self‐complete the English language outcome measures. Participants were excluded if their school had used Social Stories™ in the current or preceding school term or, the child, associated teacher or interventionist had taken part in the previous Social Stories™ feasibility study (Marshall et al., 2016).

### Randomisation and masking

Schools were randomised 1:1 using a central, computer‐based randomisation system, designed and managed by York Trials Unit. Randomisation was stratified by school type (SEN school or mainstream school) and by the number of participating children within a school (≤5 or >5 participating children) with varying block sizes (4, 6 and 8). Schools were notified of their study allocation via telephone, email or postal letter with parents/guardians receiving a letter.

Owing to the nature of the intervention, parent(s)/guardian(s), teachers and interventionists (educational professionals) were unblinded to allocation. Research assistants collecting outcome data and the main trial statistician were blinded to study allocation until final data analysis. The allocated teacher and interventionist were required to be different individuals to reduce any potential bias in the intervention delivery or completion of the outcome measures. The Data Monitoring and Ethics Committee (DMEC) had access to unblinded data throughout the study.

### Procedures

A core criterion of the intervention was to first agree a socio‐emotional goal around which the story would be set. The goal was agreed during a collaborative ‘goal setting meeting’ attended by the child's teacher, a parent/caregiver, a member of the research team and sometimes the child's teaching assistant (TA). Where possible the child was involved. Each socio‐emotional goal was required to be specific, measurable, achievable, realistic, time‐bound (SMART) and observable in the school setting, measured using a goal‐based outcome measure (see Appendix S1).

Children in the intervention group received Social Stories™ in addition to their care as usual from their school. Educational professionals (the interventionists) were pre‐selected prior to randomisation and were already employed within each school. The interventionists varied between the schools and were staff with training in the support of autistic children (e.g. a teacher, teaching assistant (TA) or special educational needs coordinator (SENCO)). In most cases, the interventionist was someone who already knew the child well and worked closely with them.

Interventionists were trained in Social Stories™ with its established 10 criteria of generation and delivery (Gray, 2010). Training used a cascade model (training the trainers; Wright et al., 2024) overseen by BW and included general autism psychoeducation as well as specific training around the use of Social Stories™. During the training session, interventionists constructed a Social Story™. Parents/guardians were also invited to attend these sessions. Story‐reading sessions were delivered by the interventionist at least six times over a 4‐week period.

Schools randomised to care as usual were asked to continue delivering any other support but to refrain from delivering any Social Stories™. At the end of the trial, schools were offered Social Stories™ training.

### Outcomes

The primary outcome was the Social Responsiveness Scale 2 (SRS‐2) at 6 months, as reported by the child's associated teacher (different from the interventionist teacher). The SRS‐2 identifies the presence and scale of social difficulty (Constantino & Gruber, 2012) and consists of 65 questions. For each question, a score from 1 to 4 (1 = *not true*, 2 = *sometimes true*, 3 = *often true*, 4 = *almost always true*) is selected that best describes the child. A *T*‐score was calculated based on the sex of the child and the person completing the form (teacher).

The following secondary outcomes were collected at baseline, 6 weeks and 6 months unless otherwise stated. Associated teachers were asked to complete the SRS‐2 (Constantino & Gruber, 2012), the goal‐based outcome measure (adapted from the Child Outcomes Research Consortium; Law & Jacob, 2013), a bespoke resource use questionnaire (baseline and 6 months), a bespoke treatment preference questionnaire (baseline) and a bespoke resource use questionnaire determining current school care/education plan interventions (baseline and 6 months). Parents completed the SRS‐2 (6 weeks; Constantino & Gruber, 2012), Parenting Stress Index short form (Abidin, 2012), health‐related quality of life (EQ‐5D‐Y (proxy1; EuroQol Group, 1990)), anxiety and depression (Revised Children Anxiety and Depression Scale short form (Chorpita, Yim, Moffitt, Umemoto, & Francis, 2000), a bespoke resource use questionnaire developed and refined during the feasibility trial capturing healthcare and non‐health and educational resource use (baseline and 6 months), and a demographic and bespoke treatment preference questionnaire (baseline). The interventionists were asked to complete demographic information including information about interventions they had previously delivered and their level of experience with autistic children, a bespoke Social Story™ session log (used after each Social Story™ session) and a bespoke sustainability questionnaire (6 weeks and 6 months). All Social Stories™ were assessed against a fidelity checklist. Additional information about the bespoke measures is provided within the Appendix S1.

### Statistical analysis

Estimates of key sample size parameters, including the variance of the primary outcome, mean cluster size, intra‐cluster correlation, attrition rate and the correlation between repeated measurements of the primary outcome, were informed by data from the feasibility study (Wright et al., 2016). In the feasibility study, the point estimate of the correlation between repeat measurements of the SRS‐2 at baseline and 6 weeks (*r* = .67, 95% CI 0.44 to 0.80) was lower than the point estimate of the correlation between measurements at baseline and 16 weeks (*r* = .83, 95% CI 0.68 to 0.91). To be conservative, the lower 95% confidence limit for the correlation between baseline and 6 weeks (*r* = .44) was used for the power calculations. For a target difference of *δ* = 3 points (slightly below the difference of 3.33 estimated from the feasibility trial data as corresponding to a clinically significant improvement), and assuming SD = 7, mean cluster size = 1.35, ICC = 0.34, correlation = 0.44 and 25% attrition, a total sample size of 278 was required to obtain 90% power for a two‐tailed test of H_0_: *δ* = 0 of size 5%. Calculations were undertaken using Stata/SE v14.0. The sample size was calculated for the primary outcome only.

The trial analyses were undertaken using Stata/MP 17.0. The primary analysis sought to estimate differences in expected SRS‐2 score at 6 months post‐randomisation, with all departures from allocated treatment being handled via a treatment policy strategy. The between group difference in expected SRS‐2 total *T*‐score at 6 months post‐randomisation was estimated using a linear mixed effect covariance pattern model, with scores at all timepoints (6 weeks and 6 months) included as an outcome. This model included fixed effects for treatment group, time point and their interaction, school SEN status, number of consented children attending the school (binary, ≤5/>5), baseline SRS‐2 total *T*‐score (linear term), age at randomisation (linear term) and sex (female/male) and school level random intercepts to capture unexplained between school variance. Correlation between repeated measurements within participants was modelled using an unstructured covariance matrix for the model residuals. Adjusted mean differences and two‐tailed 95% confidence intervals were obtained from the fitted model for each time point and presented alongside associated *p*‐values. *p*‐Values are reported primarily as a means of quantifying the divergence between the observed data and the test hypothesis for the secondary endpoints, using the conventional threshold of 0.05 as indicating statistical significance with respect to the primary endpoint.

Sensitivity analyses were undertaken to investigate the potential impact of departures from the missing at random (MAR) assumption (White, 2018; White, Carpenter, & Horton, 2018), mistimed data collection and non‐adherence using complier average causal effect (CACE) analysis, with compliance defined as receipt of at least six story sessions. For the present study, the CACE is an estimate of the average causal effect of Social Stories™ among the partially latent subgroup of participants that would have received at least six story sessions if they had (possibly counter‐to‐fact) been allocated to the intervention group. We undertook further exploratory analyses to assess whether COVID‐19‐related disruption to intervention delivery and/or follow‐up was associated with variation in treatment effects at 6 weeks and 6 months. In addition, pre‐specified subgroup analyses by teacher‐reported treatment preference, whether the child was diagnosed with a cognitive/intellectual or learning disability and whether the child was diagnosed with mental or psychological illness.

Secondary outcomes (teacher‐reported SRS‐2 total raw score, goal‐based outcome, parent‐reported SRS‐2 total *T*‐score and raw score, RCADS total score, EQ‐5D‐Y VAS and PSI total stress score) were analysed and reported in a similar manner to the primary analysis, but with the baseline values for the relevant outcome included in place of the baseline teacher‐reported SRS‐2 total *T*‐scores.

### Role of funding source

The funder of the study and the sponsor had no role in data collection, analysis interpretation, writing of the report or study design, although the funder did indicate a preference for the use of the SRS‐2 above the goal‐based outcome measure as the primary outcome due to the validity of the SRS‐2. A representative of the funder was present on the Trial Steering Committee.

## Results

**Figure 1:**
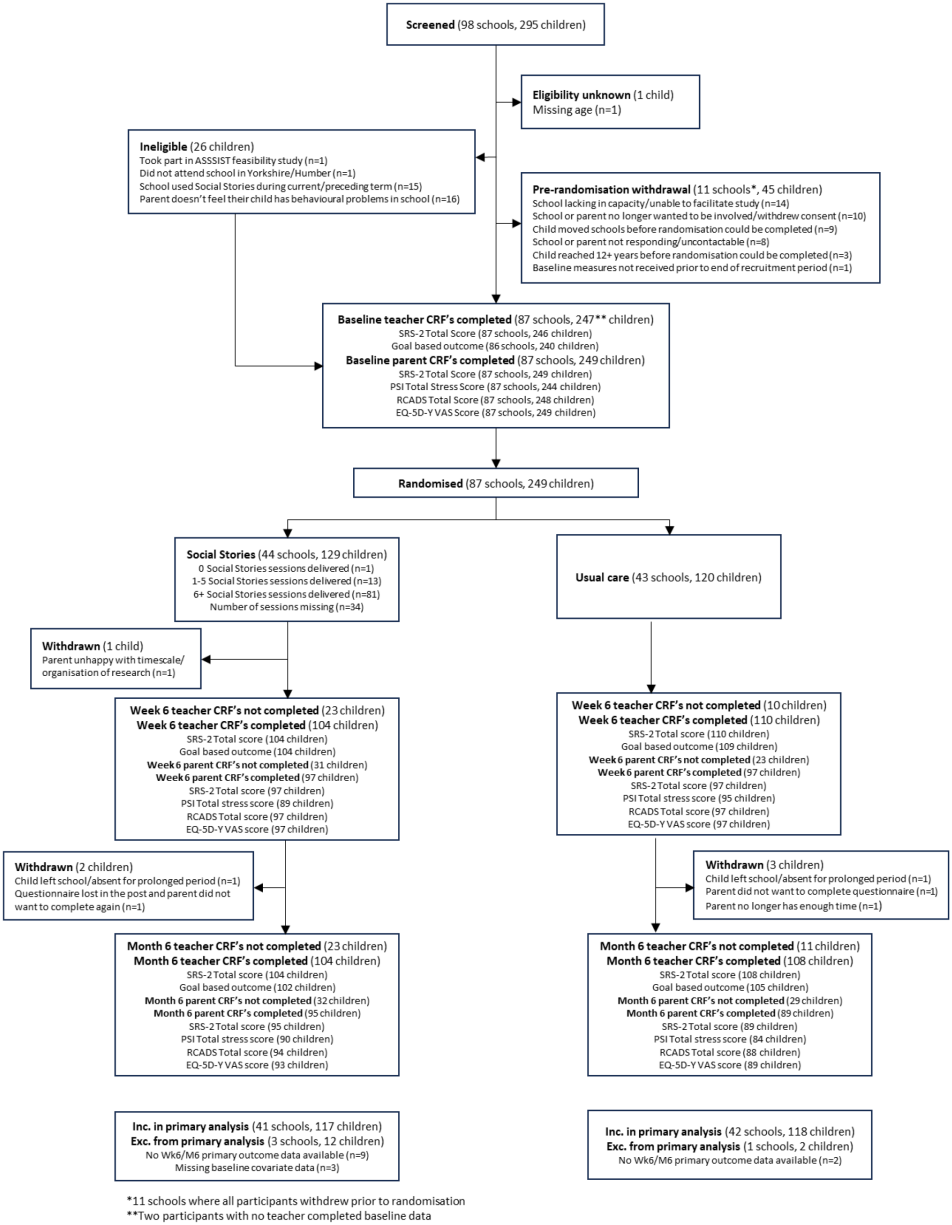
Recruitment and data collection

Between November 2018 and May 2021, 295 children across 98 schools were screened for trial eligibility (Figure 1). Out of these, 87 (89%) schools including 249 (84%) children were randomised (intervention: 44 schools with 129 children; usual care: 43 schools with 120 children). Following randomisation, five children (2%) withdrew from at least some aspect of the planned data collection. Three out of these were withdrawn from all data collection (including the teacher completed primary outcome).

Baseline characteristics were well balanced between groups. Schools had a similar average number of participating pupils and SEN status (Table S1). Children were well balanced in terms of age, sex, ethnicity, age at diagnosis and Child and Adolescent Mental Health Services (CAMHS) support (Table S2).

Of the 44 schools and 129 children allocated to Social Stories™, 36 schools (82%) and 95 (74%) children provided intervention delivery data. Of the children with available data, 81 (85%) received at least six story reading sessions, 13 (14%) received one to five sessions and one (1%) did not receive any sessions.

Based on the availability of both completed baseline and at least one follow‐up data point, 235 (94.4%) children across 83 schools were included in the pre‐specified primary analysis model. At baseline, children scored 72 points on average on the SRS‐2 indicating a moderate level of difficulty. The average SRS‐2 scores decreased in both groups over time. At 6 weeks children allocated to Social Stories™ scored on average 1.14 points lower than those who received usual care indicating slightly greater social responsiveness but this was not statistically significant (95% CI −3.35 to 1.06, *p* = .310). This difference increased slightly at 6 months to 1.61 points but again was not statistical significant (95% CI −4.18 to 0.96, *p* = .22; Tables 1, S3 and S4).

**Table 1:** Summary of the treatment effects of all primary and secondary outcomes

Outcome	Timing	Estimated difference (Intervention – Control) in expected value (95% CI^a^)	*p*‐Value^a^
Primary outcome			
SRS‐2	Week 6	−1.14 (−3.35 to 1.06)	.310
	Month 6 (primary)	−1.61 (−4.18 to 0.96)	.220
Secondary outcomes			
SRS‐2 total raw score (teacher)	Week 6	−3.37 (−9.41 to 2.67)	.274
	Month 6	−3.32 (−10.28 to 3.63)	.349
Goal‐based outcome (teacher)	Week 6	0.84 (0.14 to 1.54)	.018
	Month 6	0.97 (0.21 to 1.73)	.012
SRS‐2 total *T*‐score (parent)	Week 6	0.43 (−0.83 to 1.70)	.504
	Month 6	0.35 (−1.26 to 1.97)	.668
SRS‐2 total raw (parent)	Week 6	2.37 (−1.41 to 6.16)	.219
	Month 6	1.21 (−3.65 to 6.08)	.625
RCADS total score (parent)	Week 6	1.10 (−1.81 to 4.02)	.458
	Month 6	2.35 (−1.37 to 6.06)	.215
PSI total stress score	Week 6	−1.42 (−4.92 to 2.09)	.428
	Month 6	−1.49 (−5.43 to 2.46)	.460
EQ‐5D‐Y VAS (parent)	Week 6	−0.63 (−4.93 to 3.66)	.772
	Month 6	−1.92 (−6.70 to 2.86)	.432

^a^ Based on delta method standard errors.

The CACE analysis, found that Social Stories™ led to a −3.37 point reduction in the SRS‐2 scale (indicating higher social responsiveness) at 6 months (95% CI −6.65 to −0.10; *p* = .043; Table 2). Results from sensitivity CACE analyses are presented in the supplementary material (Tables S5–S7).

**Table 2:** SRS‐2 CACE estimates at 6 weeks and 6 months post‐randomisation from the planned analysis models

	Control (*N* = 120)	Intervention (*N* = 129)	CACE estimate (95% CI)	*p*‐Value
Week 6	110 (91.7%)	79 (61.2%)	−2.35 (−5.44 to 0.73)	.134
Month 6	108 (90.0%)	76 (58.9%)	−3.37 (−6.65 to −0.10)	.043

Children allocated to receive Social Stories™ met their socio‐emotional behavioural goal more frequently (rated by their teacher) after 6 weeks (0.84, 95% CI 0.14 to 1.54, *p* = .018) and 6 months (0.97, 95% CI 0.21 to 1.73, *p* = .012) than children who received care as usual (Table S8). Whilst goals chosen were often related to teachers identifying what they regarded as challenging behaviours, discussion between teacher, parent/carer (and child where possible) as part of the intervention usually identified distress in the child related to lack of social information (Wright et al., 2024). Topics included understanding routines, change and transitions, emotional development, social interactions and supporting classroom learning.

Table 1 shows limited evidence of important differences in any of the other secondary outcomes measuring parent (*T* score 6 weeks: 0.43, 95% CI −0.83 to 1.7; 6 months: 0.35, 95% CI −1.26 to 1.97; total raw 6 weeks: 2.37, 95% CI −1.41 to 6.16; 6 months: 1.21, 95% CI −3.65 to 6.08) and teacher social responsiveness (6 weeks: −3.37, 95% CI −9.41 to 2.67; 6 months: −3.32, 95% CI −10.28 to 3.63), children's mental health (6 weeks: 1.10, 95% CI −1.81 to 4.02; 6 months: 2.35, 95% CI −1.37 to 6.06) and well‐being (6 weeks: −0.63, 95% CI −4.93 to 3.66; 6 months: −1.92, 95% CI −6.70 to 2.86) or parental stress (6 weeks: −1.42, 05% CI −4.92 to 2.09; 6 months: −1.49, 95% CI −5.43 to 2.46; Tables S9–S13).

There was some very weak evidence that disruption to follow‐up due to COVID‐19 school closures was associated with variation in treatment effect, with the between group difference in the primary endpoint being slightly larger (in favour of Social Stories™) among those that completed outcome data collection prior to the start of the first UK lockdown on 23rd March 2020. For participants who completed data collection before school closures, the treatment effect at 6 months was −5.07 (95% CI −10.49 to 0.34) compared to −0.63 (95% CI −3.56 to 2.31) for participants who had data collected after the start of the first UK lockdown. Overall the subgroup analyses found little evidence for any interaction between treatment preference (*p* = .75), mental health (*p* = .82) and cognitive/learning difficulties (*p* = .27) and allocation.

During the trial, two serious and four non‐serious adverse events (AEs) were reported. These six AEs occurred in six individuals (i.e. at most one event per individual). Despite the slightly higher number of AEs reported in the intervention group (intervention *n* = 4), only one was deemed probably related to the intervention, this was an expected event, and there was limited evidence of any clear variation between groups.

## Discussion

This was the largest trial to date which aimed to evaluate whether Social Stories™ is a clinically effective method of improving social responsiveness in autistic children. A total of 87 schools were randomised including 249 autistic children. At baseline, children scored 72 points on average on the Social Responsiveness Scale‐2 (SRS‐2) indicating a moderate level of difficulty. The average SRS‐2 scores decreased (higher social responsiveness) in both groups at 6 weeks and 6 months, with greater reductions observed in the group allocated to Social Stories™. However, at both time points, the estimated difference (in favour of Social Stories™) was small and unlikely to be clinically important. Given SRS‐2 attempts to measure autistic symptoms it was perhaps not surprising that there was limited change. By contrast, there are statistically significant differences in the goal‐based outcome measure. Children who were allocated to Social Stories™ typically met their socio‐emotional goal with a clear measurable outcome more frequently after 6 months than children who received care as usual. However, the observed differences are smaller than the differences observed in the feasibility trial (Wright et al., 2016) and a recently published trial of humanistic counselling for adolescents with symptoms of psychological distress (Cooper et al., 2021), although goal attainment was self‐reported in the latter study in contrast to the teacher‐reported assessment used in the present study. The CACE analysis also found a clinically and statistically significant difference in favour of the intervention group at 6 months, although this estimate of the CACE may be subject to non‐negligible selection bias due to missing treatment delivery data. There were no important differences in any of the other measures assessing children's mental health and well‐being or parental stress.

To our knowledge, this was the first pragmatic trial evaluating the effectiveness and cost‐effectiveness (Wang et al., 2024) of Carol Gray's Social Stories™ for improving social responsiveness in autistic children. Response rates to the primary outcome were high. In total, 211 (84.7%) teachers provided primary outcome data at the 6‐month follow‐up point, with similar proportions in each group. Equally, despite being a typically harder to reach group, we achieved a response rate of 184 (73.9%) parents/guardians, again with similar proportions in each group. An independent Trial Steering Committee as well as a Data Monitoring and Ethics Committee provided oversight to ensure that the trial was conducted as planned and that participant safety issues were considered. A key strength of the methodology of the trial was in our approach to minimising the risk of bias. First, all participating children were recruited and underwent baseline data collection prior to cluster allocation. Second, all data were collected by blinded research assistants to reduce the risk of bias in the data gathering process. There were no cases where data were collected by someone who was not blind to allocation. Furthermore, the trial statistician remained blind to allocation until the final analysis.

However, there are several limitations that should be considered in the interpretation of our findings. A substantial portion of the study occurred whilst a worldwide pandemic was occurring. From 23rd March 2020 until the end of the school year in July 2020 and 4th January 2021 to 8th March 2021 most schools were closed. This led to large disruptions to schools with closures, staff and pupil absences and changes to the intervention construction and delivery as well as care as usual. The results of this trial should therefore be taken with caution in both potential directions. Owing to COVID‐19 restrictions, we were not able to quality assure the teacher‐reported outcome through independent observation. Given that the intervention, due to its nature, was delivered unblinded, there was some potential to introduce reporting bias into teacher ratings in favour of those receiving the intervention. We attempted to minimise this by ensuring that the associated teacher and the interventionist were different people, but some potential for bias remains. It is notoriously difficult to adequately blind the measurement of school‐based outcomes when the intervention is being delivered in school. In addition, due to the restrictions imposed as a consequence of the pandemic, the training model was updated from face‐to‐face delivery to online delivery with reliance on some level of independent learning. Although trainers asked schools whether they watched the presentation at the start of the story writing session, we are unable to accurately assess whether interventionists did watch the presentation and whether they effectively processed this information. This was much clearer during the face‐to‐face training where sessions were interactive, and more flexible use of activities could check understanding. Given the importance of the psychoeducation training, failing to access and engage with this independent learning could have had an impact on the effectiveness of the intervention. Finally, our preference would have been to use the goal‐based outcome measure as our primary outcome measure, however, a validated instrument was favoured by the funder. The challenge with a single validated outcome measure was that Social Stories™ are a complex intervention that can target a number of goals in the life of an autistic child and no single measure was likely to capture outcomes well. Furthermore, using a generic child quality of life measure has advantages related to comparability but may not be a well‐suited measure to a population of autistic children. Improved ways of considering outcome measures in pragmatic trials of complex interventions are needed.

Given the uncertainty of our findings in light of the COVID‐19 pandemic which substantially impacted the delivery of the trial post‐March 2020, we cannot definitively rule out the possibility that Social Stories™ are of greater benefit to autistic children, especially when delivered at an adequate dose and with maximum fidelity. The study reported here achieved smaller effect sizes than the feasibility study reported previously (Wright et al., 2016) and this could have been related to the effects of the pandemic noted above. Because of this, there may be value in further research around Social Stories™, particularly with regards to the impact on specific goals or sub‐goals. Indeed, a systematic review exploring the impact of Social Stories concluded that they are most beneficial when used to target specific socio‐emotional goals rather than teach general social skills (Kokina & Kern, 2010). Improved research designs should attempt to capture wider outcomes such as the value of teacher/parent interaction in understanding and planning support for the child.

Based on the evidence generated through this trial we cannot recommend Social Stories™ for the purposes of improving social skills, anxiety and /or depression, parental stress and general health in autistic children. However, we did not find any negative effects and Social Stories™ are already frequently used in schools to support autistic children and represent a low‐cost and potentially cost‐saving intervention (Wang et al., 2024). Despite limited evident impact on global social skills, Social Stories™ require dialogue between parents and school staff to better understand the specific needs of autistic children. They are viewed very positively by parents and teachers and are a positive way of delivering social information and promoting the achievement of tailored socio‐emotional goals for autistic children (Wright et al., 2024). Their usage should be at the school's discretion.

## Funding

This research was funded by NIHR HTA programme. Grant reference: NIHR HTA Programme 16/111/91.

## Conflict of interest

BW is a co‐author of ‘A Guide to Writing Social Stories™: Step‐by‐Step Guidelines for Parents and Professionals’ (Jessica Kingsley Publishers), which was provided to school staff to support their Social Stories™ training as part of the trial. All royalties are donated to Martin House Children's Hospice. CH was a member of the NIHR HTA commissioning committee (2015–2022) and Deputy Chair (2019–2022) and is now Chair of the NIHR HTA General Committee (2023–). All other authors declare no competing interests.

## Author contributions

BW and CH were chief investigators. JB, KB and CT were trial managers and JB and KB drafted the NIHR HTA final report and this article. BW and CH chaired the Trial Management Group and BW oversaw the Social Stories™ training. Trainers of intervention schools were BW, CT, KB and JB. ES, LA, OT, AT, HC, RHo, HP, and HE were research assistants responsible for data collection. LM and CW accessed and verified the data and conducted the statistical analysis. HW and SP were responsible for the economic evaluation. AS was the qualitative lead. JW, DMc, DM and DV were co‐applicants and contributed to the trial design and trial management group. RHa was the Child Oriented Mental Health Innovation Collaborative (COMIC) programme manager and contributed to the trial design. AM was a PPI representative and contributed to the trial design. All authors within the writing group reviewed and approved the contents of the paper.

## Ethical information

Ethical approval for the trial was obtained from the Health Sciences Research Governance Committee at the University of York on 02 July 2018. To allow opening of an additional recruitment stream via the NHS, ethical approval was awarded from the Health Research Authority (HRA) on 24 July 2019 and North East—York Research Ethics Committee (REC) on 23 July 2019 (19/NE/0237). Written consent was obtained from parents on behalf of children. Written consent was also obtained from all educational professionals involved.

## Supporting information


**Table S1.** Brief baseline characteristics (stratification factors and cluster sizes) of randomised schools.
**Table S2.** Parent‐reported demographics of randomised participants.
**Table S3.** SRS‐2T‐score (teacher reported) model parameter estimates, standard errors, test‐statistics, *p*‐values, variance components and model fit.
**Table S4.** SRS‐2 raw score (teacher reported) analysis model parameter estimates, standard errors, test‐statistics, *p*‐values, variance components and model fit.
**Table S5.** CACE analysis of SRS‐2T‐scores at 6 weeks (teacher reported) parameter estimates, standard errors, test‐statistics, *p*‐values, variance components and model fit from second‐stage regression.
**Table S6.** CACE analysis of SRS‐2T‐scores at 6 months (teacher reported) parameter estimates, standard errors, test‐statistics, *p*‐values, variance components and model fit from second‐stage regression.
**Table S7.** Complier average causal effect (CACE) estimates for the SRS‐2 scores at 6 months post‐randomisation under various assumptions about the missing compliance data
**Table S8.** Goal‐based outcome analysis model parameter estimates, standard errors, test‐statistics, *p*‐values, variance components and model fit.
**Table S9.** SRS‐2T‐score (parent reported) analysis model parameter estimates, standard errors, test‐statistics, *p*‐values, variance components and model fit.
**Table S10.** SRS‐2 raw score (parent reported) analysis model parameter estimates, standard errors, test‐statistics, *p*‐values, variance components and model fit.
**Table S11.** RCADS total score (parent reported) analysis model parameter estimates, standard errors, test‐statistics, *p*‐values, variance components and model fit.
**Table S12.** PSI total stress score (parent reported) analysis model parameter estimates, standard errors, test‐statistics, *p*‐values, variance components and model fit.
**Table S13.** EQ‐5D‐Y VAS score (parent reported) analysis model parameter estimates, standard errors, test‐statistics, *p*‐values, variance components and model fit.
**Appendix S1.** Bespoke outcome measures.

## Data Availability

The study protocol is publicly available. Anonymised participant‐level data for the quantitative clinical effectiveness evaluation and data dictionary, are available from the Open Science Framework repository via https://osf.io/fjz2v/. Analysis code and the final approved version of the statistical analysis plan will also be made available via this repository.
