# Supplementary material for: Autism Spectrum Social Stories in Schools Trial 2 (ASSSIST‐2): a pragmatic randomised controlled trial of the Social Stories™ intervention to address the social and emotional health of autistic children in UK primary schools
Source: Child Adolesc Ment Health. 2024 Dec 17;30(1):4–12. doi: 10.1111/camh.12740 (PMC11754701; doi:10.1111/camh.12740)
Supplement: Supplementary file 1 — Table S1. Brief baseline characteristics (stratification factors and cluster sizes) of randomised schools. Table S2. Parent‐reported demographics of randomised participants. Table S3. SRS‐2T‐score (teacher reported) model parameter estimates, standard errors, test‐statistics, p‐values, variance components and model fit. Table S4. SRS‐2 raw score (teacher reported) analysis model parameter estimates, standard errors, test‐statistics, p‐values, variance components and model fit. Table S5. CACE analysis of SRS‐2T‐scores at 6 weeks (teacher reported) parameter estimates, standard errors, test‐statistics, p‐values, variance components and model fit from second‐stage regression. Table S6. CACE analysis of SRS‐2T‐scores at 6 months (teacher reported) parameter estimates, standard errors, test‐statistics, p‐values, variance components and model fit from second‐stage regression. Table S7. Complier average causal effect (CACE) estimates for the SRS‐2 scores at 6 months post‐randomisation under various assumptions about the missing compliance data Table S8. Goal‐based outcome analysis model parameter estimates, standard errors, test‐statistics, p‐values, variance components and model fit. Table S9. SRS‐2T‐score (parent reported) analysis model parameter estimates, standard errors, test‐statistics, p‐values, variance components and model fit. Table S10. SRS‐2 raw score (parent reported) analysis model parameter estimates, standard errors, test‐statistics, p‐values, variance components and model fit. Table S11. RCADS total score (parent reported) analysis model parameter estimates, standard errors, test‐statistics, p‐values, variance components and model fit. Table S12. PSI total stress score (parent reported) analysis model parameter estimates, standard errors, test‐statistics, p‐values, variance components and model fit. Table S13. EQ‐5D‐Y VAS score (parent reported) analysis model parameter estimates, standard errors, test‐statistics, p‐values, variance components and model f [file CAMH-30-4-s001.docx]

**Table S1.** Brief baseline characteristics (stratification factors and cluster sizes) of randomised schools

|  | **Control (N = 43)** | **Intervention (N = 44)** | **Total (N = 87)** |
| --- | --- | --- | --- |
| **School SEN status stratum, n (%)** |  |  |  |
| Non-SEN | 39 (90.7) | 40 (90.9) | 79 (90.8) |
| SEN | 4 (9.3) | 4 (9.1) | 8 (9.2) |
| **Number of participants stratum, n (%)** |  |  |  |
| ≤5 | 33 (76.7) | 35 (79.5) | 68 (78.2) |
| >5 | 10 (23.3) | 9 (20.5) | 19 (21.8) |
| **Randomisation stratum, n (%)** |  |  |  |
| Non-SEN and ≤5 | 30 (69.8) | 32 (72.7) | 62 (71.3) |
| SEN and ≤5 | 3 (7.0) | 3 (6.8) | 6 (6.9) |
| Non-SEN and >5 | 9 (20.9) | 8 (18.2) | 17 (19.5) |
| SEN and >5 | 1 (2.3) | 1 (2.3) | 2 (2.3) |
| **Cluster size (as randomised)** |  |  |  |
| N | 43 | 44 | 87 |
| Mean (SD) | 2.8 (1.9) | 2.9 (2.0) | 2.9 (2.0) |
| Median (Q1, Q3) | 2.0 (1.0, 4.0) | 2.0 (2.0, 4.0) | 2.0 (1.0, 4.0) |
| Min, Max | 1.0, 9.0 | 1.0, 11.0 | 1.0, 11.0 |

**Table S2.** Parent reported demographics of randomised participants

|  | **Control (N = 120)** | **Intervention (N = 129)** | **Total (N = 249)** |
| --- | --- | --- | --- |
| **Age (years)** |  |  |  |
| N | 120 | 129 | 249 |
| Mean (SD) | 8.6 (1.8) | 8.5 (1.7) | 8.5 (1.7) |
| Median (Q1, Q3) | 8.9 (7.2, 10.0) | 8.7 (7.2, 9.8) | 8.7 (7.2, 9.9) |
| Min, Max | 4.9, 11.4 | 4.5, 11.9 | 4.5, 11.9 |
| **Sex, n (%)** |  |  |  |
| Male | 90 (75.0) | 95 (73.6) | 185 (74.3) |
| Female | 30 (25.0) | 34 (26.4) | 64 (25.7) |
| **Ethnicity, n (%)** |  |  |  |
| White - British | 103 (85.8) | 107 (82.9) | 210 (84.3) |
| White - Irish | 1 (0.8) | 0 (0.0) | 1 (0.4) |
| White - Gypsy^1^/Traveller | 0 (0.0) | 1 (0.8) | 1 (0.4) |
| White - Other | 2 (1.7) | 6 (4.7) | 8 (3.2) |
| Black - African | 1 (0.8) | 1 (0.8) | 2 (0.8) |
| Asian - Indian | 2 (1.7) | 0 (0.0) | 2 (0.8) |
| Asian - Pakistani | 1 (0.8) | 5 (3.9) | 6 (2.4) |
| Asian - Bangladeshi | 1 (0.8) | 0 (0.0) | 1 (0.4) |
| Asian - Chinese | 0 (0.0) | 1 (0.8) | 1 (0.4) |
| Asian - Other | 0 (0.0) | 2 (1.6) | 2 (0.8) |
| Mixed - White and Black Caribbean | 1 (0.8) | 2 (1.6) | 3 (1.2) |
| Mixed - White and Asian | 2 (1.7) | 2 (1.6) | 4 (1.6) |
| Mixed - Other | 3 (2.5) | 2 (1.6) | 5 (2.0) |
| Other - Other | 1 (0.8) | 0 (0.0) | 1 (0.4) |
| Prefer not to say | 2 (1.7) | 0 (0.0) | 2 (0.8) |
| **Autism diagnosis confirmed by^2^** |  |  |  |
| Psychiatrist | 8 (6.7) | 19 (14.7) | 27 (10.8) |
| Clinical psychologist | 50 (41.7) | 49 (38.0) | 99 (39.8) |
| Speech and Language Therapist | 30 (25.0) | 36 (27.9) | 66 (26.5) |
| Educational psychologist | 19 (15.8) | 18 (14.0) | 37 (14.9) |
| Paediatrician | 64 (53.3) | 71 (55.0) | 135 (54.2) |
| Other | 16 (13.3) | 15 (11.6) | 31 (12.4) |
| **Autism diagnosis part of multidisciplinary assessment?, n (%)** |  |  |  |
| Yes | 116 (96.7) | 119 (92.2) | 235 (94.4) |
| No | 3 (2.5) | 10 (7.8) | 13 (5.2) |
| Missing | 1 (0.8) | 0 (0.0) | 1 (0.4) |
| **Child's age at time of Austism diagnosis (years)** |  |  |  |
| N | 120 | 129 | 249 |
| Mean (SD) | 5.5 (2.2) | 5.2 (2.1) | 5.3 (2.1) |
| Median (Q1, Q3) | 5.0 (4.0, 7.0) | 5.0 (4.0, 7.0) | 5.0 (4.0, 7.0) |
| Min, Max | 2.0, 10.0 | 2.0, 10.0 | 2.0, 10.0 |
| **Child receiving CAMHS support?, n (%)** |  |  |  |
| Yes | 10 (8.3) | 11 (8.5) | 21 (8.4) |
| No | 110 (91.7) | 118 (91.5) | 228 (91.6) |
| **Comorbidities^2^** |  |  |  |
| Physical health problems | 31 (25.8) | 33 (25.6) | 64 (25.7) |
| Mental health or psychological problems | 16 (13.3) | 15 (11.6) | 31 (12.4) |
| Developmental problems or learning difficulties | 18 (15.0) | 17 (13.2) | 35 (14.1) |
| Cognitive problems or learning disability | 4 (3.3) | 8 (6.2) | 12 (4.8) |
| Genetic or chromosomal problems | 5 (4.2) | 2 (1.6) | 7 (2.8) |
| ^1^ This term was used through self-identification ^2^Possibly more than one category per individual | | | |

**Table S3.** SRS-2 T-score (teacher reported) model parameter estimates, standard errors, test-statistics, p-values, variance components and model fit.

| **Fixed Effects** (p* < 0.001) | **Coef.** | **Std. err.** | **z** | **P>\|z\|** | **95% conf. interval** | |
| --- | --- | --- | --- | --- | --- | --- |
| Allocation |  |  |  |  |  |  |
| Social Stories | -1.14 | 1.13 | -1.01 | 0.310 | -3.35 | 1.06 |
|  |  |  |  |  |  |  |
| Time point |  |  |  |  |  |  |
| Month 6 | -0.18 | 0.86 | -0.21 | 0.834 | -1.87 | 1.51 |
|  |  |  |  |  |  |  |
| Allocation $\times$ Time point |  |  |  |  |  |  |
| Social Stories $\times$ Month 6 | -0.47 | 1.26 | -0.37 | 0.709 | -2.93 | 1.99 |
|  |  |  |  |  |  |  |
| SEN status |  |  |  |  |  |  |
| SEN | 2.76 | 1.80 | 1.53 | 0.126 | -0.77 | 6.30 |
|  |  |  |  |  |  |  |
| School cluster size |  |  |  |  |  |  |
| >5 children | -0.58 | 1.07 | -0.55 | 0.584 | -2.68 | 1.51 |
|  |  |  |  |  |  |  |
| Baseline score | 0.71 | 0.04 | 15.91 | <0.001 | 0.62 | 0.80 |
|  |  |  |  |  |  |  |
| Age | -0.09 | 0.27 | -0.34 | 0.730 | -0.63 | 0.44 |
|  |  |  |  |  |  |  |
| Sex |  |  |  |  |  |  |
| Female | 1.75 | 1.08 | 1.62 | 0.104 | -0.36 | 3.86 |
|  |  |  |  |  |  |  |
| Intercept | 20.29 | 4.06 | 5.00 | <0.001 | 12.33 | 28.25 |
|  |  |  |  |  |  |  |
| **Variance components** | **Coef.** | **Std. err.** |  |  | **95% conf. interval** | |
| School random intercept | 3.75 | 3.42 |  |  | 0.63 | 22.44 |
|  |  |  |  |  |  |  |
| Week 6 variance | 53.81 | 6.19 |  |  | 42.95 | 67.42 |
|  |  |  |  |  |  |  |
| Month 6 variance | 76.95 | 8.08 |  |  | 62.64 | 94.52 |
|  |  |  |  |  |  |  |
| Week 6 $\times$ Month 6 covariance | 26.50 | 5.58 |  |  | 15.57 | 37.44 |
|  |  |  |  |  |  |  |
| **Treatment effects** | **Coef.** | **Std. err.** | **z** | **P>\|z\|** | **95% conf. interval** | |
| Week 6 | -1.14 | 1.13 | -1.01 | 0.310 | -3.35 | 1.06 |
|  |  |  |  |  |  |  |
| Month 6 | -1.61 | 1.31 | -1.23 | 0.220 | -4.18 | 0.96 |

* p-value for test of the hypothesis that all fixed effects are zero

**Table S4.** SRS-2 raw score (teacher reported) analysis model parameter estimates, standard errors, test-statistics, p-values, variance components and model fit.

| **Fixed Effects** (p* < 0.001) | **Coef.** | **Std. err.** | **z** | **P>\|z\|** | **95% conf. interval** | |
| --- | --- | --- | --- | --- | --- | --- |
| Allocation |  |  |  |  |  |  |
| Social Stories | -3.37 | 3.08 | -1.09 | 0.274 | -9.41 | 2.67 |
|  |  |  |  |  |  |  |
| Time point |  |  |  |  |  |  |
| Month 6 | -1.38 | 2.36 | -0.59 | 0.558 | -6.00 | 3.24 |
|  |  |  |  |  |  |  |
| Allocation $\times$ Time point |  |  |  |  |  |  |
| Social Stories $\times$ Month 6 | 0.05 | 3.44 | 0.01 | 0.989 | -6.69 | 6.78 |
|  |  |  |  |  |  |  |
| SEN status |  |  |  |  |  |  |
| SEN | 6.70 | 4.92 | 1.36 | 0.173 | -2.94 | 16.34 |
|  |  |  |  |  |  |  |
| School cluster size |  |  |  |  |  |  |
| >5 children | -1.92 | 2.90 | -0.66 | 0.507 | -7.61 | 3.76 |
|  |  |  |  |  |  |  |
| Baseline score | 0.73 | 0.04 | 16.48 | <0.001 | 0.64 | 0.81 |
|  |  |  |  |  |  |  |
| Age | -0.34 | 0.75 | -0.46 | 0.649 | -1.81 | 1.13 |
|  |  |  |  |  |  |  |
| Sex |  |  |  |  |  |  |
| Female | -1.17 | 2.91 | -0.40 | 0.688 | -6.88 | 4.54 |
|  |  |  |  |  |  |  |
| Intercept | 26.56 | 8.11 | 3.28 | 0.001 | 10.67 | 42.45 |
| **Variance components** | **Coef.** | **Std. err.** |  |  | **95% conf. interval** | |
| School random intercept | 24.53 | 25.55 |  |  | 3.18 | 189.00 |
|  |  |  |  |  |  |  |
| Week 6 variance | 415.00 | 47.77 |  |  | 331.18 | 520.04 |
|  |  |  |  |  |  |  |
| Month 6 variance | 571.82 | 60.02 |  |  | 465.49 | 702.44 |
|  |  |  |  |  |  |  |
| Week 6 $\times$ Month 6 covariance | 202.34 | 42.35 |  |  | 119.33 | 285.36 |
|  |  |  |  |  |  |  |
| **Treatment effects** | **Coef.** | **Std. err.** | **z** | **P>\|z\|** | **95% conf. interval** | |
| Week 6 | -3.37 | 3.08 | -1.09 | 0.274 | -9.41 | 2.67 |
|  |  |  |  |  |  |  |
| Month 6 | -3.32 | 3.55 | -0.94 | 0.349 | -10.28 | 3.63 |

*p-value for test of the hypothesis that all fixed effects are zero

**Table S5.** CACE analysis of SRS-2 T-scores at 6 weeks (teacher reported) parameter estimates, standard errors, test-statistics, p-values, variance components and model fit from second stage regression.

| **Fixed Effects** (p* < 0.001) | **Coef.** | **Std. err.** | **z** | **P>\|z\|** | **95% conf. interval** | |
| --- | --- | --- | --- | --- | --- | --- |
| Allocation |  |  |  |  |  |  |
| Social Stories | -2.35 | 1.57 | -1.50 | 0.134 | -5.44 | 0.73 |
|  |  |  |  |  |  |  |
| SEN status |  |  |  |  |  |  |
| SEN | 2.25 | 2.20 | 1.03 | 0.305 | -2.05 | 6.56 |
|  |  |  |  |  |  |  |
| School cluster size |  |  |  |  |  |  |
| >5 children | -0.24 | 1.35 | -0.18 | 0.860 | -2.89 | 2.41 |
|  |  |  |  |  |  |  |
| Baseline score | 0.78 | 0.05 | 15.06 | <0.001 | 0.68 | 0.88 |
|  |  |  |  |  |  |  |
| Age | -0.03 | 0.33 | -0.09 | 0.924 | -0.67 | 0.61 |
|  |  |  |  |  |  |  |
| Sex |  |  |  |  |  |  |
| Female | 1.21 | 1.29 | 0.94 | 0.348 | -1.32 | 3.73 |
|  |  |  |  |  |  |  |
| Intercept | 14.79 | 4.73 | 3.13 | 0.002 | 5.52 | 24.06 |
| **Variance components** | **Coef.** |  |  |  |  | |
| School random intercept | 7.89 |  |  |  |  |  |
|  |  |  |  |  |  |  |
| Residual variance | 54.32 |  |  |  |  |  |
|  |  |  |  |  |  |  |

*p-value for test of the hypothesis that all fixed effects are zero

**Table S6.** CACE analysis of SRS-2 T-scores at 6 months (teacher reported) parameter estimates, standard errors, test-statistics, p-values, variance components and model fit from second stage regression.

| **Fixed Effects** (p* < 0.001) | **Coef.** | **Std. err.** | **z** | **P>\|z\|** | **95% conf. interval** | |
| --- | --- | --- | --- | --- | --- | --- |
| Allocation |  |  |  |  |  |  |
| Social Stories | -3.37 | 1.67 | -2.02 | 0.043 | -6.65 | -0.10 |
|  |  |  |  |  |  |  |
| SEN status |  |  |  |  |  |  |
| SEN | 4.05 | 2.49 | 1.63 | 0.104 | -0.83 | 8.93 |
|  |  |  |  |  |  |  |
| School cluster size |  |  |  |  |  |  |
| >5 children | -0.25 | 1.45 | -0.17 | 0.864 | -3.10 | 2.60 |
|  |  |  |  |  |  |  |
| Baseline score | 0.55 | 0.06 | 8.65 | <0.001 | 0.42 | 0.67 |
|  |  |  |  |  |  |  |
| Age | 0.04 | 0.39 | 0.09 | 0.929 | -0.73 | 0.80 |
|  |  |  |  |  |  |  |
| Sex |  |  |  |  |  |  |
| Female | 2.33 | 1.50 | 1.56 | 0.119 | -0.60 | 5.27 |
|  |  |  |  |  |  |  |
| Intercept | 30.28 | 5.73 | 5.28 | <0.001 | 19.05 | 41.51 |
| **Variance components** | **Coef.** |  |  |  |  | |
| School random intercept | 4.72 |  |  |  |  |  |
|  |  |  |  |  |  |  |
| Residual variance | 78.20 |  |  |  |  |  |
|  |  |  |  |  |  |  |

*p-value for test of the hypothesis that all fixed effects are zero

**Table S7.** Complier Average Causal Effect (CACE) estimates for the SRS-2 scores at 6 months post-randomisation under various assumptions about the missing compliance data

| **Assumption** | **CACE estimate (95% CI)** | **p-value** |
| --- | --- | --- |
| Compliance data are missing completely at random | -3.37 (-6.65 to -0.10) | 0.043 |
| Intervention group participants missing compliance data are compliers (i.e. received at least 6 story sessions)) | -1.87 (-4.68 to 0.93) | 0.110 |
| Intervention group participants missing compliance data are non-compliers (i.e. received less than 6 story sessions)) | -2.59 (-6.27 to 1.08) | 0.167 |

**Table S8.** Goal-based outcome analysis model parameter estimates, standard errors, test-statistics, p-values, variance components and model fit.

| **Fixed Effects** (p* < 0.001) | **Coef.** | **Std. err.** | **z** | **P>\|z\|** | **95% conf. interval** | |
| --- | --- | --- | --- | --- | --- | --- |
| Allocation |  |  |  |  |  |  |
| Social Stories | 0.843 | 0.357 | 2.37 | 0.018 | 0.14 | 1.54 |
|  |  |  |  |  |  |  |
| Time point |  |  |  |  |  |  |
| Month 6 | 0.91 | 0.25 | 3.64 | <0.001 | 0.42 | 1.40 |
|  |  |  |  |  |  |  |
| Allocation $\times$ Time point |  |  |  |  |  |  |
| Social Stories $\times$ Month 6 | 0.13 | 0.36 | 0.35 | 0.723 | -0.58 | 0.84 |
|  |  |  |  |  |  |  |
| SEN status |  |  |  |  |  |  |
| SEN | -0.47 | 0.56 | -0.84 | 0.400 | -1.56 | 0.62 |
|  |  |  |  |  |  |  |
| School cluster size |  |  |  |  |  |  |
| >5 children | 0.14 | 0.34 | 0.40 | 0.688 | -0.53 | 0.81 |
|  |  |  |  |  |  |  |
| Baseline score | 0.23 | 0.07 | 3.34 | 0.001 | 0.10 | 0.37 |
|  |  |  |  |  |  |  |
| Age | 0.05 | 0.08 | 0.64 | 0.523 | -0.11 | 0.21 |
|  |  |  |  |  |  |  |
| Sex |  |  |  |  |  |  |
| Female | 0.26 | 0.31 | 0.86 | 0.389 | -0.34 | 0.86 |
|  |  |  |  |  |  |  |
| Intercept | 3.23 | 0.75 | 4.32 | <0.001 | 1.77 | 4.70 |
| **Variance components** | **Coef.** | **Std. err.** |  |  | **95% conf. interval** | |
| School random intercept | 0.61 | 0.38 |  |  | 0.18 | 2.05 |
|  |  |  |  |  |  |  |
| Week 6 variance | 4.58 | 0.55 |  |  | 3.62 | 5.79 |
|  |  |  |  |  |  |  |
| Month 6 variance | 5.58 | 0.63 |  |  | 4.47 | 6.95 |
|  |  |  |  |  |  |  |
| Week 6 $\times$ Month 6 covariance | 1.96 | 0.47 |  |  | 1.03 | 2.89 |
|  |  |  |  |  |  |  |
| **Treatment effects** | **Coef.** | **Std. err.** | **z** | **P>\|z\|** | **95% conf. interval** | |
| Week 6 | 0.84 | 0.36 | 2.37 | 0.018 | 0.14 | 1.54 |
|  |  |  |  |  |  |  |
| Month 6 | 0.97 | 0.39 | 2.51 | 0.012 | 0.21 | 1.73 |

*p-value for test of the hypothesis that all fixed effects are zero

**Table S9.** SRS-2 T-score (parent reported) analysis model parameter estimates, standard errors, test-statistics, p-values, variance components and model fit.

| **Fixed Effects** (p* < 0.001) | **Coef.** | **Std. err.** | **z** | **P>\|z\|** | **95% conf. interval** | |
| --- | --- | --- | --- | --- | --- | --- |
| Allocation |  |  |  |  |  |  |
| Social Stories | 0.43 | 0.65 | 0.67 | 0.504 | -0.83 | 1.70 |
|  |  |  |  |  |  |  |
| Time point |  |  |  |  |  |  |
| Month 6 | 0.18 | 0.52 | 0.35 | 0.726 | -0.84 | 1.20 |
|  |  |  |  |  |  |  |
| Allocation $\times$ Time point |  |  |  |  |  |  |
| Social Stories $\times$ Month 6 | -0.08 | 0.73 | -0.11 | 0.916 | -1.51 | 1.36 |
|  |  |  |  |  |  |  |
| SEN status |  |  |  |  |  |  |
| SEN | 1.57 | 1.07 | 1.47 | 0.142 | -0.53 | 3.66 |
|  |  |  |  |  |  |  |
| School cluster size |  |  |  |  |  |  |
| >5 children | 0.33 | 0.64 | 0.52 | 0.602 | -0.92 | 1.58 |
|  |  |  |  |  |  |  |
| Baseline score | 0.76 | 0.04 | 20.38 | <0.001 | 0.69 | 0.84 |
|  |  |  |  |  |  |  |
| Age | 0.08 | 0.18 | 0.42 | 0.673 | -0.28 | 0.43 |
|  |  |  |  |  |  |  |
| Sex |  |  |  |  |  |  |
| Female | 7.74 | 0.70 | 11.10 | <0.001 | 6.37 | 9.10 |
|  |  |  |  |  |  |  |
| Intercept | 12.81 | 3.53 | 3.62 | <0.001 | 5.88 | 19.73 |
| **Variance components** | **Coef.** | **Std. err.** |  |  | **95% conf. interval** | |
| School random intercept | <0.01 | <0.01 |  |  | 0 | - |
|  |  |  |  |  |  |  |
| Week 6 variance | 20.70 | 2.11 |  |  | 16.96 | 25.28 |
|  |  |  |  |  |  |  |
| Month 6 variance | 32.66 | 3.42 |  |  | 26.60 | 40.10 |
|  |  |  |  |  |  |  |
| Week 6 $\times$ Month 6 covariance | 15.04 | 2.19 |  |  | 10.75 | 19.33 |
|  |  |  |  |  |  |  |
| **Treatment effects** | **Coef.** | **Std. err.** | **z** | **P>\|z\|** | **95% conf. interval** | |
| Week 6 | 0.43 | 0.65 | 0.67 | 0.504 | -0.83 | 1.70 |
|  |  |  |  |  |  |  |
| Month 6 | 0.35 | 0.83 | 0.43 | 0.668 | -1.26 | 1.97 |

*p-value for test of the hypothesis that all fixed effects are zero

**Table S10.** SRS-2 raw score (parent reported) analysis model parameter estimates, standard errors, test-statistics, p-values, variance components and model fit.

| **Fixed Effects** (p* < 0.001) | **Coef.** | **Std. err.** | **z** | **P>\|z\|** | **95% conf. interval** | |
| --- | --- | --- | --- | --- | --- | --- |
| Allocation |  |  |  |  |  |  |
| Social Stories | 2.37 | 1.93 | 1.23 | 0.219 | -1.41 | 6.16 |
|  |  |  |  |  |  |  |
| Time point |  |  |  |  |  |  |
| Month 6 | 0.59 | 1.60 | 0.37 | 0.711 | -2.54 | 3.72 |
|  |  |  |  |  |  |  |
| Allocation $\times$ Time point |  |  |  |  |  |  |
| Social Stories $\times$ Month 6 | -1.16 | 2.25 | -0.52 | 0.606 | -5.58 | 3.26 |
|  |  |  |  |  |  |  |
| SEN status |  |  |  |  |  |  |
| SEN | 2.58 | 3.17 | 0.81 | 0.416 | -3.64 | 8.80 |
|  |  |  |  |  |  |  |
| School cluster size |  |  |  |  |  |  |
| >5 children | 0.54 | 1.89 | 0.28 | 0.777 | -3.18 | 4.25 |
|  |  |  |  |  |  |  |
| Baseline score | 0.82 | 0.04 | 21.28 | <0.001 | 0.74 | 0.89 |
|  |  |  |  |  |  |  |
| Age | 0.07 | 0.53 | 0.13 | 0.896 | -0.98 | 1.12 |
|  |  |  |  |  |  |  |
| Sex |  |  |  |  |  |  |
| Female | 2.61 | 2.04 | 1.28 | 0.200 | -1.39 | 6.61 |
|  |  |  |  |  |  |  |
| Intercept | 14.53 | 6.65 | 2.18 | 0.029 | 1.49 | 27.57 |
| **Variance components** | **Coef.** | **Std. err.** |  |  | **95% conf. interval** | |
| School random intercept | <0.01 | <0.01 |  |  | 0 | - |
|  |  |  |  |  |  |  |
| Week 6 variance | 185.06 | 18.91 |  |  | 151.47 | 226.10 |
|  |  |  |  |  |  |  |
| Month 6 variance | 294.00 | 31.14 |  |  | 238.89 | 361.83 |
|  |  |  |  |  |  |  |
| Week 6 $\times$ Month 6 covariance | 129.13 | 19.65 |  |  | 90.63 | 167.64 |
|  |  |  |  |  |  |  |
| **Treatment effects** | **Coef.** | **Std. err.** | **z** | **P>\|z\|** | **95% conf. interval** | |
| Week 6 | 2.37 | 1.93 | 1.23 | 0.219 | -1.41 | 6.16 |
|  |  |  |  |  |  |  |
| Month 6 | 1.21 | 2.48 | 0.49 | 0.625 | -3.65 | 6.08 |

*p-value for test of the hypothesis that all fixed effects are zero

**Table S11.** RCADS total score (parent reported) analysis model parameter estimates, standard errors, test-statistics, p-values, variance components and model fit.

| **Fixed Effects** (p* < 0.001) | **Coef.** | **Std. err.** | **z** | **P>\|z\|** | **95% conf. interval** | |
| --- | --- | --- | --- | --- | --- | --- |
| Allocation |  |  |  |  |  |  |
| Social Stories | 1.10 | 1.49 | 0.74 | 0.458 | -1.81 | 4.02 |
|  |  |  |  |  |  |  |
| Time point |  |  |  |  |  |  |
| Month 6 | -0.45 | 1.24 | -0.36 | 0.716 | -2.88 | 1.98 |
|  |  |  |  |  |  |  |
| Allocation $\times$ Time point |  |  |  |  |  |  |
| Social Stories $\times$ Month 6 | 1.25 | 1.76 | 0.71 | 0.478 | -2.19 | 4.68 |
|  |  |  |  |  |  |  |
| SEN status |  |  |  |  |  |  |
| SEN | -1.82 | 2.44 | -0.75 | 0.455 | -6.60 | 2.95 |
|  |  |  |  |  |  |  |
| School cluster size |  |  |  |  |  |  |
| >5 children | -1.30 | 1.44 | -0.91 | 0.365 | -4.13 | 1.52 |
|  |  |  |  |  |  |  |
| Baseline score | 0.86 | 0.03 | 26.88 | <0.001 | 0.80 | 0.92 |
|  |  |  |  |  |  |  |
| Age | -0.05 | 0.42 | -0.12 | 0.901 | -0.88 | 0.77 |
|  |  |  |  |  |  |  |
| Sex |  |  |  |  |  |  |
| Female | 1.37 | 1.56 | 0.88 | 0.381 | -1.70 | 4.44 |
|  |  |  |  |  |  |  |
| Intercept | 5.62 | 3.79 | 1.48 | 0.139 | -1.82 | 13.06 |
| **Variance components** | **Coef.** | **Std. err.** |  |  | **95% conf. interval** | |
| School random intercept | <0.01 | <0.01 |  |  | <0.01 | <0.01 |
|  |  |  |  |  |  |  |
| Week 6 variance | 109.01 | 15.96 |  |  | 81.82 | 145.24 |
|  |  |  |  |  |  |  |
| Month 6 variance | 168.67 | 21.14 |  |  | 131.94 | 215.63 |
|  |  |  |  |  |  |  |
| Week 6 $\times$ Month 6 covariance | 72.96 | 26.18 |  |  | 21.64 | 124.28 |
|  |  |  |  |  |  |  |
| **Treatment effects** | **Coef.** | **Std. err.** | **z** | **P>\|z\|** | **95% conf. interval** | |
| Week 6 | 1.10 | 1.49 | 0.74 | 0.458 | -1.81 | 4.02 |
|  |  |  |  |  |  |  |
| Month 6 | 2.35 | 1.89 | 1.24 | 0.215 | -1.36 | 6.06 |

*p-value for test of the hypothesis that all fixed effects are zero

**Table S12.** PSI total stress score (parent reported) analysis model parameter estimates, standard errors, test-statistics, p-values, variance components and model fit.

| **Fixed Effects** (p* < 0.001) | **Coef.** | **Std. err.** | **z** | **P>\|z\|** | **95% conf. interval** | |
| --- | --- | --- | --- | --- | --- | --- |
| Allocation |  |  |  |  |  |  |
| Social Stories | -1.42 | 1.79 | -0.79 | 0.428 | -4.92 | 2.09 |
|  |  |  |  |  |  |  |
| Time point |  |  |  |  |  |  |
| Month 6 | -0.42 | 1.44 | -0.29 | 0.772 | -3.24 | 2.41 |
|  |  |  |  |  |  |  |
| Allocation $\times$ Time point |  |  |  |  |  |  |
| Social Stories $\times$ Month 6 | -.072 | 2.04 | -0.04 | 0.972 | -4.08 | 3.93 |
|  |  |  |  |  |  |  |
| SEN status |  |  |  |  |  |  |
| SEN | 0.30 | 2.72 | 0.11 | 0.912 | -5.03 | 5.64 |
|  |  |  |  |  |  |  |
| School cluster size |  |  |  |  |  |  |
| >5 children | -0.18 | 1.64 | -0.11 | 0.911 | -3.39 | 3.03 |
|  |  |  |  |  |  |  |
| Baseline score | 0.88 | 0.04 | 22.10 | <0.001 | 0.80 | 0.96 |
|  |  |  |  |  |  |  |
| Age | -0.16 | 0.47 | -0.35 | 0.728 | -1.07 | 0.75 |
|  |  |  |  |  |  |  |
| Sex |  |  |  |  |  |  |
| Female | 0.37 | 1.76 | 0.21 | 0.834 | -3.09 | 3.83 |
|  |  |  |  |  |  |  |
| Intercept | 14.50 | 6.09 | 2.38 | 0.017 | 2.56 | 26.45 |
| **Variance components** | **Coef.** | **Std. err.** |  |  | **95% conf. interval** | |
| School random intercept | <0.01 | <0.01 |  |  | <0.01 | 0.51 |
|  |  |  |  |  |  |  |
| Week 6 variance | 147.48 | 16.96 |  |  | 117.72 | 184.76 |
|  |  |  |  |  |  |  |
| Month 6 variance | 177.85 | 21.15 |  |  | 140.87 | 224.52 |
|  |  |  |  |  |  |  |
| Week 6 $\times$ Month 6 covariance | 80.54 | 14.79 |  |  | 51.56 | 109.52 |
|  |  |  |  |  |  |  |
| **Treatment effects** | **Coef.** | **Std. err.** | **z** | **P>\|z\|** | **95% conf. interval** | |
| Week 6 | -1.42 | 1.79 | -0.79 | 0.428 | -4.92 | 2.09 |
|  |  |  |  |  |  |  |
| Month 6 | -1.49 | 2.01 | -0.74 | 0.460 | -5.43 | 2.46 |

*p-value for test of the hypothesis that all fixed effects are zero

**Table S13.** EQ-5D-Y VAS score (parent reported) analysis model parameter estimates, standard errors, test-statistics, p-values, variance components and model fit.

| **Fixed Effects** (p* < 0.001) | **Coef.** | **Std. err.** | **z** | **P>\|z\|** | **95% conf. interval** | |
| --- | --- | --- | --- | --- | --- | --- |
| Allocation |  |  |  |  |  |  |
| Social Stories | -0.63 | 2.19 | -0.29 | 0.772 | -4.93 | 3.66 |
|  |  |  |  |  |  |  |
| Time point |  |  |  |  |  |  |
| Month 6 | 1.81 | 1.73 | 1.04 | 0.297 | -1.59 | 5.19 |
|  |  |  |  |  |  |  |
| Allocation $\times$ Time point |  |  |  |  |  |  |
| Social Stories $\times$ Month 6 | -1.29 | 2.46 | -0.52 | 0.601 | -6.09 | 3.52 |
|  |  |  |  |  |  |  |
| SEN status |  |  |  |  |  |  |
| SEN | 2.22 | 3.35 | 0.66 | 0.508 | -4.34 | 8.78 |
|  |  |  |  |  |  |  |
| School cluster size |  |  |  |  |  |  |
| >5 children | 0.05 | 2.00 | 0.03 | 0.978 | -3.87 | 3.98 |
|  |  |  |  |  |  |  |
| Baseline score | 0.42 | 0.05 | 7.72 | <0.001 | 0.31 | 0.53 |
|  |  |  |  |  |  |  |
| Age | -0.23 | 0.57 | -0.41 | 0.682 | -1.35 | 0.88 |
|  |  |  |  |  |  |  |
| Sex |  |  |  |  |  |  |
| Female | 2.02 | 2.19 | 0.92 | 0.357 | -2.28 | 6.31 |
|  |  |  |  |  |  |  |
| Intercept | 47.66 | 7.05 | 6.76 | <0.001 | 33.85 | 61.48 |
| **Variance components** | **Coef.** | **Std. err.** |  |  | **95% conf. interval** | |
| School random intercept | <0.01 | <0.01 |  |  | 0 | - |
|  |  |  |  |  |  |  |
| Week 6 variance | 237.04 | 24.72 |  |  | 193.22 | 290.79 |
|  |  |  |  |  |  |  |
| Month 6 variance | 279.40 | 30.40 |  |  | 225.74 | 345.81 |
|  |  |  |  |  |  |  |
| Week 6 $\times$ Month 6 covariance | 127.72 | 23.16 |  |  | 82.32 | 173.11 |
|  |  |  |  |  |  |  |
| **Treatment effects** | **Coef.** | **Std. err.** | **z** | **P>\|z\|** | **95% conf. interval** | |
| Week 6 | -0.63 | 2.19 | -0.29 | 0.772 | -4.93 | 3.66 |
|  |  |  |  |  |  |  |
| Month 6 | -1.92 | 2.44 | -0.79 | 0.432 | -6.70 | 2.86 |

*p-value for test of the hypothesis that all fixed effects are zero

**Appendix S1.** Bespoke outcome measures

**Bespoke goal-based outcome measure-**Completed at baseline, 6 week and 6 month follow up by the associated teacher (validated with blinded observation of 20% of participants in a classroom setting by research assistants at 6 weeks and at 6 months).

At baseline the Associated Teacher set a behavioural goal for the participating child (with assistance from the parent/guardian, teaching assistant and child where possible). The Associated Teacher rated how close the child was to meeting this particular goal at that time using our bespoke goal-based measure. The scale ranged from 1-10, where 0 indicates that the child was meeting their goal none of the time and 10 indicated that the child was meeting their goal all of the time. Teachers were asked to complete the bespoke goal-based measure at both the 6 week and 6-month follow-up to rate how close the child is to meeting the goal at these time points. To validate this measure, at the 6 week and 6-month follow-up data collection points, we aimed for 20% of the children to be observed in a classroom setting by a member of the research team (blinded to intervention allocation) and for the researcher to complete the same goal-based measure as the Associated Teacher. Consent to these observations was optional and participants were randomly selected from those who opted in. We took a pragmatic approach to the observations; some behavioural goals are focused on behaviour at playtime, and the behaviour in question would in all likelihood not display in the classroom. In these cases, we tried to arrange to observe the child in a playtime setting. Similarly, in some cases the goals relate to behaviour that is not easily observable or able to be observed, in which case we did not complete the observation of this participant (e.g. a goal about toileting). As noted in the main paper, owing to COVID-19 restrictions, we were not able to quality assure the teacher reported outcome through independent observation from March 2020.

**Bespoke resource use questionnaire-Completed** at baseline and 6 months follow up only by parents/guardians

At baseline and at the 6 month follow up parents/guardians were asked to complete a bespoke resource use questionnaire, which captures the healthcare and non-health resource implications (including costs in the education sector, and costs of productivity loss and out-of-pocket expenditures for parents) attributable to the child’s difficulties due to their condition.

**Bespoke treatment preference questionnaire–** Completed at baseline only by parent/guardians and associate teachers

This is a single visual analogue scale [0, 100], where 0 indicates strong preference for usual care/support, 100 indicates strong preference for Social Stories and 50 indicates indifference/no preference.

**Bespoke Social Story™ session log–** Completed by interventionists (educational professionals) after each Social Story™ session

A Social Story™ session log used to record frequency, length and times of Social Story^TM^ sessions. In addition, the log provides space to record any problems or adverse events that occurred during delivery.

**A bespoke sustainability questionnaire**– Completed by interventionists at the 6 week and 6 month follow ups

A bespoke sustainability questionnaire for interventionists to record how many times they have used the intervention with the same child and/or different children and whether they have trained other staff in how to write and deliver Social Stories.
